# Digital Health Literacy as a Predictor of Awareness, Engagement, and Use of a National Web-Based Personal Health Record: Population-Based Survey Study

**DOI:** 10.2196/35772

**Published:** 2022-09-16

**Authors:** Christina Cheng, Emma Gearon, Melanie Hawkins, Crystal McPhee, Lisa Hanna, Roy Batterham, Richard H Osborne

**Affiliations:** 1 Centre for Global Health and Equity School of Health Sciences Swinburne University of Technology Hawthorn Australia; 2 Department of Epidemiology and Preventive Medicine Monash University Melbourne Australia; 3 Beyondblue Melbourne Australia; 4 School of Health and Social Development Faculty of Health Deakin University Geelong Australia; 5 Faculty of Public Health Thammasat University Bangkok Thailand

**Keywords:** eHealth, mobile health, mHealth, health literacy, health equity, electronic health records, vulnerable populations, disadvantaged populations

## Abstract

**Background:**

Web-based personal health records (PHRs) have the potential to improve the quality, accuracy, and timeliness of health care. However, the international uptake of web-based PHRs has been slow. Populations experiencing disadvantages are less likely to use web-based PHRs, potentially widening health inequities within and among countries.

**Objective:**

With limited understanding of the predictors of community uptake and use of web-based PHR, the aim of this study was to identify the predictors of awareness, engagement, and use of the Australian national web-based PHR, My Health Record (MyHR).

**Methods:**

A population-based survey of adult participants residing in regional Victoria, Australia, was conducted in 2018 using telephone interviews. Logistic regression, adjusted for age, was used to assess the relationship among digital health literacy, health literacy, and demographic characteristics, and the 3 dependent variables of MyHR: awareness, engagement, and use. Digital health literacy and health literacy were measured using multidimensional tools, using all 7 scales of the eHealth Literacy Questionnaire and 4 out of the 9 scales of the Health Literacy Questionnaire.

**Results:**

A total of 998 responses were analyzed. Many elements of digital health literacy were strongly associated with MyHR awareness, engagement, and use. A 1-unit increase in each of the 7 eHealth Literacy Questionnaire scales was associated with a 2- to 4-fold increase in the odds of using MyHR: *using technology to process health information* (odds ratio [OR] 4.14, 95% CI 2.34-7.31), *understanding of health concepts and language* (OR 2.25, 95% CI 1.08-4.69), *ability to actively engage with digital services* (OR 4.44, 95% CI 2.55-7.75), *feel safe and in control* (OR 2.36, 95% CI 1.43-3.88), *motivated to engage with digital services* (OR 4.24, 95% CI 2.36-7.61), *access to digital services that work* (OR 2.49, 95% CI 1.32-4.69), and *digital services that suit individual needs* (OR 3.48, 95% CI 1.97-6.15)*.* The Health Literacy Questionnaire scales of health care support, actively managing health, and social support were also associated with a 1- to 2-fold increase in the odds of using MyHR. Using the internet to search for health information was another strong predictor; however, older people and those with less education were less likely to use MyHR.

**Conclusions:**

This study revealed strong and consistent patterns of association between digital health literacy and the use of a web-based PHR. The results indicate potential actions for promoting PHR uptake, including improving digital technology and skill experiences that may improve digital health literacy and willingness to engage in web-based PHR. Uptake may also be improved through more responsive digital services, strengthened health care, and better social support. A holistic approach, including targeted solutions, is needed to ensure that web-based PHR can realize its full potential to help reduce health inequities.

## Introduction

### Background

Digital technologies have enabled the storage of personal health information in a web-based environment where people can keep track of and access their health records as needed [1-4]. The World Health Organization considers a national web-based personal health record (PHR) an important component of universal health coverage, given its potential to improve the quality, accuracy, and timeliness of health care [5]. In 2016, half (47%) of the member states responding to a survey indicated that they had introduced a national web-based PHR system [5,6].

In 2012, Australia, a country with universal health coverage, rolled out a national web-based PHR system, My Health Record (MyHR), aiming to provide Australians with *safer, faster and more efficient* health care [7,8]. MyHR is a secure web-based summary of a person’s health information, allowing people to control and share personal health information with their health care providers anytime and anywhere, thereby improving communication between clinicians and patients. The system is available in more than 18 languages and is enabled for people with low vision and blindness to ensure access to people with various needs [9]. However, MyHR uptake was slow. Only approximately 21% (5.2 million out of 24.6 million) of Australia’s total population signed up for MyHR in 2017, after 5 years of rollout [10]. In 2018, the Australian Government announced that MyHR would become an opt-out system, meaning that people would automatically be enrolled in MyHR unless they elected not to be during the opt-out period between July 2018 and January 2019 [11].

The slow uptake of web-based PHRs is not unique to Australia [1]. A study in the United States reported that only 20% of survey participants used web-based PHRs [12]. After an investment of £8 million (approximately US $11 million) in a web-based PHR in the United Kingdom in 2007, the system HealthSpace was abandoned in 2011 owing to low adoption. The postevaluation report commented that the lack of health literacy and digital literacy in some users might be one of the reasons for nonadoption [13].

### Digital Health Literacy

Digital health literacy (also called eHealth literacy) can be one of the deciding factors when determining whether to use a digital health system [14-16]. Digital health literacy refers to an individual’s “ability to seek, find, understand, and appraise health information from electronic sources and apply the knowledge gained to addressing or solving a health problem” [14]. This concept has continued to evolve with the ever-changing digital landscape since it was first conceived in 2006. Nevertheless, it is grounded in health literacy, which is defined as “people’s knowledge, confidence, and comfort, which accumulate through daily activities, social interactions, and across generations, to access, understand, appraise, remember, and use information about health and health care for the health and wellbeing of themselves and those around them” (World Health Organization, unpublished data, September 2022).

PHRs have the potential to reduce health inequities [17,18]. However, this potential may be derailed by the digital divide [2,3], which refers to inequitable access, use, and outcomes of technology use among subgroups of society, because people with higher income or education are more likely to have better access or skills to use technology than other groups, such as culturally diverse minorities, rural residents, or people with lower income or education [19-22]. These groups are also less likely to use web-based PHRs [3,23] and face more digital health literacy challenges [24-27]. Hence, well-intentioned efforts to reduce health inequities must be implemented with care to ensure that they do not worsen health disparities [2,3,23]. Understanding the predictors of using web-based PHRs is an essential first step to avoid this pitfall.

To date, research on the predictors of web-based PHR uptake is limited. Only a few studies could be identified, and they mostly focused on age, education, health status, computer skills, and experiences in web-based information seeking [28-33]. Only one study has specifically examined digital health literacy [33]. Given the limited understanding of the predictors of uptake and use of web-based PHR, the aim of this study was to determine the predictors of awareness, engagement, and use of the Australian personal web-based health record MyHR during the MyHR opt-out period, with a focus on digital health literacy. Regarding predictors, this study referred to the statistical procedure used to identify factors associated with PHR uptake and use, not as an indication of the causal relationship with these factors.

## Methods

### Study Design

This study was part of a larger study conducted in the city of Ballarat and the surrounding regional area in Victoria, Australia [34]. Victoria is the second most populous state in Australia, with a population of approximately 6.7 million as of June 2021 [35]. The city of Ballarat is situated in the regional area of north-central Victoria. This area was selected as it was a trial site for the early implementation of the MyHR [36] and chosen in partnership with the Australian Digital Health Agency, the commonwealth entity established to oversee the operation and evolution of Australian digital health capability [37]. The aim of the larger study was to generate insights into how to maximize the uptake and use of MyHR and other digital technologies. The study was an application of the Optimizing Health Literacy and Access (Ophelia) process, which involved identifying local needs and then using a co-design approach to engage stakeholders (consumers, practitioners, and managers) to generate fit-for-purpose solutions through insights from local wisdom [38]. This paper presents the findings from the needs assessment phase of the study.

### Data Collection

A computer-assisted telephone interview (CATI) survey was administered from October 1, 2018, to October 31, 2018, to participants from the general population. Participants were eligible for inclusion if they were aged >18 years, able to complete a telephone survey in English, and resided in the Ballarat Goldfields region of Victoria, Australia. There were no quotas for age or sex. The interviews were conducted by a contract research company (Strahan Research Pty Ltd). The interviewer team underwent specialized training with the project team. A pilot phase was conducted, with the responses reviewed before the formal implementation of the survey. Each interview started with a description of the project requirements and the consent process, followed by the survey. Only respondents who consented to participate were included in this study. Each interview lasted approximately 17 minutes. The completed CATI surveys were deidentified before being provided to the project team.

To ensure the sample was stratified by socioeconomic position, it was drawn using systematic random sampling: all postcodes of the region were ordered by an area-level marker of socioeconomic position based on the Index of Relative Socioeconomic Disadvantage, an index that summarizes the economic and social conditions of people within an area [39]. A database of both landline telephone and mobile phone numbers was matched to the postal areas, and the sample was drawn using a random start fixed interval sampling technique. The fixed interval was calculated by dividing the total population of the Ballarat Goldfields region by the desired sample size of 1000.

### Ethics Approval

Ethics approval was obtained from the Deakin University Human Research Ethics Committee (HEAG-H 157_2018).

### Survey Instruments

To examine the possible predictors of awareness, engagement, and use of MyHR, digital health literacy assessment, demographic data, and use of health services data were collected. Digital health literacy was assessed using the eHealth Literacy Questionnaire (eHLQ) [40], complemented by the Health Literacy Questionnaire (HLQ) [41] to provide context for the eHLQ results. The CATI survey consisted of all 7 scales of the eHLQ and 4 scales from the HLQ, followed by questions about the participants’ demographics, health status, use of physical and digital health services, and experience of MyHR.

The eHLQ and HLQ were developed using a grounded validity-driven approach [42] to assess the multidimensional concepts of digital health literacy and health literacy. Both tools, at construction and initial validity testing, were found to be psychometrically robust [40,41], with later studies presenting acceptable to strong psychometric properties when the tools were used in different contexts [43-51]. The eHLQ consists of 35 items, with 7 scales representing the 7 dimensions of digital health literacy (see Multimedia Appendix 1 for scale definitions):

1. Using technology to process health information
2. Understanding of health concepts and language
3. Ability to actively engage with digital services
4. Feel safe and in control
5. Motivated to engage with digital services
6. Access to digital services that work
7. Digital services that suit individual needs

A 4-point response option of strongly disagree, disagree, agree, and strongly agree was used in the eHLQ. Scale scores were calculated by averaging the item scores within each scale with equal weighting, yielding 7 scale scores, each with a score range of 1 to 4 [40].

The full HLQ consists of 44 items across 9 scales [41] (see Multimedia Appendix 1 for scale definitions). However, to reduce the length of the telephone interviews, only 4 scales were used in this survey (in italics as follows):

1. *Feeling understood and supported by health care providers*
2. Having sufficient information to manage my health
3. *Actively managing my health*
4. *Social support for health*
5. Appraisal of health information
6. Ability to actively engage with health care providers
7. *Navigating the health care system*
8. Ability to find good information
9. Understand health information well enough to know what to do

Scales 1 and 4 of the HLQ reflect a social orientation that is positive in managing one’s health, and a PHR is a potentially valuable tool for this, and scale 1 also reflects the quality of communication and trust in health care providers, which is regarded as potentially important as health care providers are likely to be a key source of information for people about whether they use the MyHR. Scales 3 and 7 reflect general engagement with health and health care and are expected to provide context regarding a person’s level of interest in MyHR.

The HLQ was subjected to rigorous validity testing in the initial validation study. The unidimensionality of each scale was established with evidence of satisfactory fit for each of the nine 1-factor models, and composite reliability of each scale ranged from 0.77 to 0.90 [41]. Validity evidence for the English version was further confirmed in subsequent validation studies in Australia [43,47,52,53], the English version in other contexts [54] and elsewhere [44-46,55-57], confirming that the HLQ scales can be used independently to measure different dimensions of health literacy.

Similar to the eHLQ, scales 1, 3, and 4 of the HLQ also use the 4-point response options of strongly disagree to strongly agree. For items of scale 7, a 5-point response option of cannot do or always difficult, usually difficult, sometimes difficult, usually easy, and always easy is used. The calculation of scale scores is the same as that of the eHLQ, except for HLQ scale 7, which has a score range of 1 to 5.

For the assessment of demographic background, age was measured as a continuous variable (years); sex was measured as female or male; highest educational attainment was measured in 4 categories (did not complete secondary school; completed secondary school; trade, apprenticeship, certificate, or diploma; and university); number of chronic health conditions was a discrete variable, calculated as the sum of any of the 8 conditions—asthma, arthritis, anxiety, cancer, cardiovascular disease or heart problems, chronic pain, depression, and diabetes (no condition; 1 condition; and 2 or more conditions); self-rated health was measured on a discrete scale with 6 options, ranging from excellent to very poor; number of contacts with a health professional in the past 12 months was measured in 4 categories (≥13 times; 7-12 times; 2-6 times; and 0-1 time); and use of the internet to search for health-related information in the past 12 months was measured as a binary variable (yes or no).

**Figure 1:**
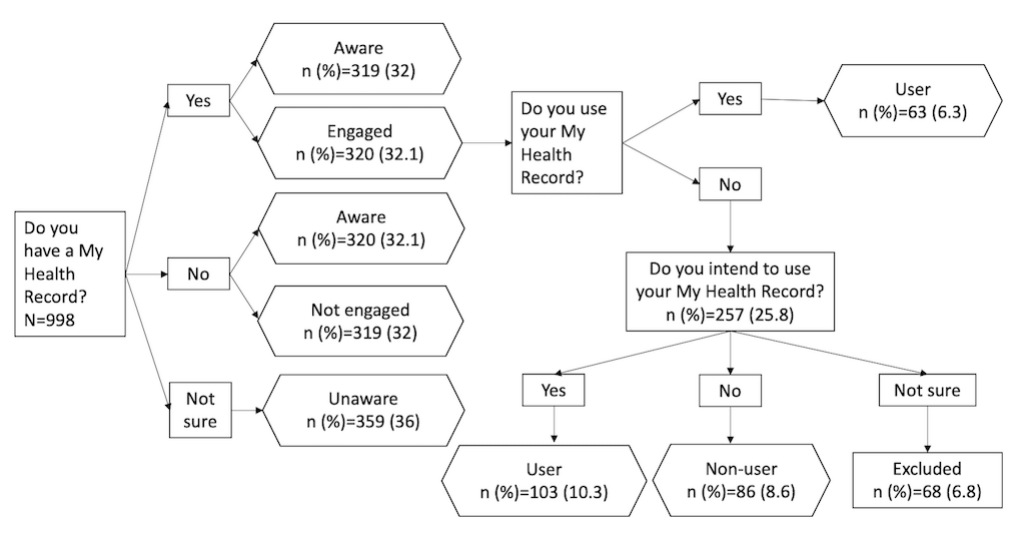
Flow diagram for identifying My Health Record awareness (aware or not aware), engagement (engaged or not engaged), and use or intention to use (user or nonuser; N=998).

The 3 variables—MyHR awareness, MyHR engagement, and MyHR use or intention to use—were determined by 3 questions in the survey. MyHR awareness was measured in the total sample using the question *Do you have a My Health Record?* (yes, no, or not sure). Participants who answered not sure were classified as *unaware*, and those who responded either yes or no were classified as *aware*, because it was a clear indication that they were aware of the existence of MyHR. For MyHR engagement, participants who answered yes to the aforementioned question were classified as *engaged*, and those who answered no, as *not engaged*. MyHR use was measured in the *engaged* subpopulation, who were asked, *Do you use your My Health Record?* (yes and no). Participants who answered yes were categorized as *user*, whereas participants who responded with a no were directed to the question, *Do you intend to use My Health Record?* (yes, no, or not sure). Participants who responded yes to this final question were classified as *intend to use* and were categorized as *user*. Those who responded that they did not intend to use MyHR were categorized as *nonuser*. To ensure that the variable gave a clear signal, *engaged* participants who chose the *not sure* option were excluded from analysis. Figure 1 shows the flow diagram for identifying awareness (aware or unaware), engagement (engaged or not engaged), and use or intention to use (user or nonuser).

### Statistical Analysis

As this was an exploratory study, a sample size of 1000 was chosen to provide ample power to detect small to moderate differences across target sex, age, education, and other subgroups (ie, 0.1-0.2–unit differences among subgroups on the 4-point response range on the eHLQ scales, with a power of at least 0.8, α of .05, and SD of 0.7), conservatively based on sample characteristics reported in the validity testing paper by Kayser et al [40].

The data were analyzed using Stata (version 15; StataCorp) [58]. Descriptive statistics of demographics and mean and 95% CIs were calculated for eHLQ and HLQ scores. The demographics of the sample were compared with those of the population of the Ballarat Goldfields region of the Western Victoria Primary Health Network (WVPHN) [59] to determine the representativeness of the sample. Primary health networks are independent primary health care organizations established throughout Australia by the Australian Government in 2015 to reform the primary health care system using a patient-oriented approach to medical services in their regions. The Ballarat Goldfields region of Victoria is part of the WVPHN, covering 21 areas in the western district of Victoria, Australia.

Logistic regression, adjusted for age, was performed to assess the relationship among independent variables, including demographic characteristics, digital health literacy, and health literacy, and the 3 dependent variables of MyHR: awareness, engagement, and use. Data collected via the eHLQ and HLQ were treated as continuous variables. The study was explorative, and no hypothesis was established, with independent variables for analysis selected based on factors plausibly associated with engagement with digital health technologies.

To ensure that the assumptions of the logistic regression were met, independent variables were collapsed as required to ensure that there were 20 or more people in each stratum. As such, the self-rated health variable was collapsed into 5 categories for all 3 dependent variables, with poor and very poor combined. The number of contacts with a health professional was collapsed into 3 categories for MyHR engagement and 2 categories for MyHR use. Education was collapsed into 3 categories, and self-rated health was 2 categories for MyHR use.

## Results

### Sample Characteristics

A total of 2839 calls were made and 1000 responses were recorded, resulting in a response rate of 35.2%. Of the 1000 CATI survey responses, 2 participants were found to be ineligible for the survey because of age, leaving the data to 998 participants for analysis. For these 998 participants, there were no missing data for any of the variables. Table 1 presents the descriptive statistics of the final sample. Compared with the Ballarat Goldfields region population, the sample had a similar proportion of people who were identified as Aboriginal or Torres Strait Islander (18/998, 1.8% for the survey compared with 2204/157,472, 1.4%) and a similar proportion of women (536/998, 53.7% for the survey compared with 80,594/157,472, 51.18%). However, the survey sample had a higher proportion of people aged 55 to 70 years (384/998, 38.5% for the survey vs 31,344/157,472, 19.9%) and >70 years (441/998, 41.2% for the survey vs 20,091/157,472, 12.76%), and a higher proportion of people with a university education (307/998, 30.8% for the survey vs 26,770/157,472, 17%; see Multimedia Appendix 2).

Approximately half of the sample (462/998, 46.3%) were male participants, and 79.7% (796/998) were aged >55 years. Approximately one-fourth of the sample (272/998, 27.3%) were living with 1 chronic condition, and 14.1% (141/998) reported having 2 or more of the 8 chronic conditions presented, with arthritis (144/998, 14.4%) and cardiovascular disease or heart problems (129/998, 12.9%) being the most commonly reported. More than half of the sample (578/998, 57.9%) reported having used the internet in the past year to search for health-related information (Table 1). Approximately two-thirds of the participants (639/998, 64%) were aware of MyHR, and 32.1% (320/998) were engaged with MyHR. However, only 6.3% (63/998) were current users of MyHR, and 10.3% (103/998) of the participants indicated that they intended to use the system (Figure 1).

For digital health literacy, participants generally disagreed (for mean score of <2.5 on a scale, ranging from 1=strongly disagree to 4=strongly agree) that they were using technology to process health information (eHLQ scale 1), had the ability to engage with digital services (eHLQ scale 3), were motivated to engage with digital services (eHLQ scale 5), and had digital services that suit their needs (eHLQ scale 7). However, they reported a generally good understanding of health concepts (eHLQ scale 2). For health literacy, participants agreed that they had good social (HLQ scale 4) and health care support (HLQ scale 1) and were actively managing their health (HLQ scale 3; Table 1).

**Table 1:** Participant characteristics (N=998).

Characteristics			Value
**Sex, n (%)**			
	Female	536 (53.7)	
	Male	462 (46.3)	
**Age (years), n (%)**			
	18 to <35	33 (3.3)	
	35 to <55	169 (16.9)	
	55 to <75	562 (56.3)	
	≥75	234 (23.4)	
Spoke English at home, n (%)			8 (0.8)
Aboriginal or Torres Strait Islander, n (%)			18 (1.8)
**Education level, n (%)**			
	Completed primary school or below	91 (9.1)	
	Did not complete secondary school	218 (21.8)	
	Completed secondary school	136 (13.6)	
	Trade, apprenticeship, certificate, or diploma	246 (24.6)	
	University	307 (30.8)	
**Reported long-standing conditions^a^, n (%)**			
	Arthritis	144 (14.4)	
	Asthma	42 (4.2)	
	Cancer	53 (5.3)	
	Cardiovascular disease or heart problems	129 (12.9)	
	Diabetes	84 (8.4)	
	Anxiety	29 (2.9)	
	Depression	45 (4.5)	
	Chronic pain	87 (8.7)	
**Number of long-standing conditions reported^b^, n (%)**			
	0	585 (58.6)	
	1	272 (27.3)	
	2	102 (10.2)	
	3 or more	39 (3.9)	
**Number of contacts with a health professional in the past 12 months, n (%)**			
	12 or more	269 (27)	
	7 to 11	196 (19.6)	
	2 to 6	446 (44.7)	
	Once	61 (6.1)	
	Not at all	26 (2.6)	
**Self-rated health, n (%)**			
	Excellent	170 (17)	
	Very good	270 (27.1)	
	Good	293 (29.4)	
	Fair	165 (16.5)	
	Poor	76 (7.6)	
	Very poor	24 (2.4)	
**Whether internet is used to search for health-related information, n (%)**			
	Yes	578 (57.9)	
	No	420 (42.1)	
**eHealth Literacy Questionnaire scales (score range 1 to 4), mean (95% CI)**			
	1. Using technology to process health information	2.34 (2.31-2.38)	
	2. Understanding of health concepts and language	2.93 (2.91-2.96)	
	3. Ability to actively engage with digital services	2.46 (2.42-2.49)	
	4. Feel safe and in control	2.59 (2.56-2.63)	
	5. Motivated to engage with digital services	2.40 (2.37-2.44)	
	6. Access to digital services that work	2.51 (2.48-2.54)	
	7. Digital services that suit individual needs	2.39 (2.35-2.42)	
**Health Literacy Questionnaire scales (score range 1 to 4), mean (95% CI)**			
	1. Feeling understood and supported by health care providers	3.20 (3.10-3.20)	
	3. Actively managing my health	3.02 (2.99-3.05)	
	4. Social support for health	3.05 (3.02-3.08)	
**Health Literacy Questionnaire (score range 1 to 5), mean (95% CI) scale**			
	7. Navigating the health care system	3.95 (3.90-3.99)	

^a^Participants might select more than one condition.

^b^Number based on long-standing conditions selected by participants (range 0-8).

### Predictors of MyHR Awareness, Engagement, and Use or Intention to Use

#### Awareness

Among all independent variables tested, digital health literacy was a strong predictor of MyHR awareness (Table 2 and Multimedia Appendix 3). With the exception of the eHLQ scale 2*, Understanding of health concepts and language*, a 1-unit increase in each of the other 6 eHLQ scales was associated with a 1.28- to 1.99-fold increase in the odds of MyHR awareness, with scale 6*, Access to digital services that work,* being the strongest. However, the 4 HLQ scales, representing health literacy, were not predictors of MyHR awareness.

For demographic factors, only sex and internet use to search for health-related information in the past 12 months were moderately strong predictors of MyHR awareness. Being female (odds ratio [OR] 1.44, 95% CI 1.11-1.87) and using the internet for health information (OR 1.52, 95% CI 1.15-2.01) were associated with higher odds of MyHR awareness compared with being male and not using the internet (Multimedia Appendix 3).

**Table 2:** Logistic regression predicting likelihood of My Health Record awareness, engagement, and use or intention to use, as measured by the eHealth Literacy Questionnaire and Health Literacy Questionnaire Scales.

Scale used			Awareness (aware n=639; unaware n=359)				Engagement (engaged n=320; not engaged n=319)					Use or intention to use (user n=166; nonuser n=86)		
			Odds ratio (95% CI)		*P* value		Odds ratio (95% CI)		*P* value		Odds ratio (95% CI)			*P* value
**eHealth Literacy Questionnaire scales**														
	1. Using technology to process health information	1.77 (1.42-2.22)		<.001		1.81 (1.35-2.42)		.001		4.14 (2.34-7.31)			<.001	
	2. Understanding of health concepts and language	1.28 (0.92-1.77)		.14		2.62 (1.70-4.03)		<.001		2.25 (1.08-4.69)			.03	
	3. Ability to actively engage with digital services	1.53 (1.25-1.89)		<.001		2.12 (1.60-2.81)		<.001		4.44 (2.55-7.75)			<.001	
	4. Feel safe and in control	1.47 (1.17-1.85)		.001		1.61 (1.20-2.14)		.001		2.36 (1.43-3.88)			.001	
	5. Motivated to engage with digital services	1.75 (1.40-2.19)		<.001		2.00 (1.48-2.71)		<.001		4.24 (2.36-7.61)			<.001	
	6. Access to digital services that work	1.99 (1.51-2.64)		<.001		1.90 (1.33-2.70)		<.001		2.49 (1.32-4.69)			.005	
	7. Digital services that suit individual needs	1.63 (1.30-2.04)		<.001		1.89 (1.40-2.55)		<.001		3.48 (1.97-6.15)			<.001	
**Health Literacy Questionnaire scales**														
	1. Feeling understood and supported by health care providers	1.15 (0.90-1.48)		.26		1.63 (1.19-2.22)		.002		1.89 (1.10-3.27)			.02	
	3. Actively managing my health	0.89 (0.66-1.21)		.46		1.23 (0.87-1.80)		.24		2.28 (1.18-4.38)			.01	
	4. Social support for health	1.02 (0.78-1.33)		.89		1.74 (1.25-2.42)		.001		2.10 (1.15-3.84)			.02	
	7. Navigating the health care system	1.10 (0.92-1.31)		.29		1.15 (0.93-1.42)		.20		1.24 (0.87-1.75)			.23	

#### Engagement

All 7 dimensions of digital health literacy, as assessed by the eHLQ, were strongly associated with MyHR engagement (Table 2 and Multimedia Appendix 4) compared with the other independent variables. A 1-unit increase on each scale was associated with a 1.61- to 2.62-fold increase in the odds of MyHR engagement. Unlike MyHR awareness, in which scale 2, *Understanding of health concepts and language,* was not a predictor; this dimension demonstrated the strongest association with engagement among all the digital health literacy dimensions.

Although the 4 health literacy dimensions assessed by the HLQ were not predictors of awareness, HLQ scale 1, *Feeling understood and supported by health care providers,* and scale 4, *Social support for health,* were significant predictors of MyHR engagement. A 1-unit increase in each scale was associated with 1.63-fold and 1.74-fold increase in the odds of engagement (Table 2 and Multimedia Appendix 4).

The use of the internet to search for health-related information continues to be an important predictor of MyHR engagement, in addition to awareness. Other strong predictors of engagement included age and the number of chronic conditions. Younger people were significantly more likely to engage with the MyHR, with every 10-year grouping increase being associated with a 20% reduction in the odds of MyHR engagement (OR 0.98, 95% CI 0.97-0.99). Having 2 or more chronic diseases or conditions was associated with a 1.88-fold increase in the odds of engagement compared with having no chronic conditions (OR 1.88, 95% CI 1.16-3.07; Multimedia Appendix 4).

#### Use or Intention to Use

Digital health literacy was again a strong predictor of MyHR use or intention to use (Table 2 and Multimedia Appendix 5). A 1-unit increase in each of the eHLQ scales was associated with a 2.25- to 4.44-fold increase in the odds of using or intending to use MyHR, with scale 3, *Ability to engage with digital services,* being notably strong, followed by scale 5, *Motivated to engage with digital services*, and scale 1, *Using technology to process health information*.

Health literacy was also a significant predictor of MyHR use. A 1-unit increase in HLQ scale 1, *Feeling understood and supported by health care providers*, scale 3, *Actively managing my health,* and scale 4, *Social support for health,* was associated with a 1.89-, 2.28-, and 2.1-fold increase in the odds of using or intending to use MyHR.

Being female (OR 1.78, 95% CI 1.05-3.00), having a university education (OR 2.48, 95% CI 1.23-5.02), and using the internet to search for health-related information in the past year (OR 2.96, 95% CI 1.64-5.37) were more likely to use or intend to use MyHR compared with being male, not completing secondary school, or not using the internet to search for health-related information (Multimedia Appendix 5).

## Discussion

### Principal Findings

This study used a population-based survey to explore the predictors of awareness, engagement, and use of a national web-based PHR, MyHR, using multidimensional measures of health literacy. Digital health literacy was strongly and consistently associated with MyHR awareness, engagement, and use. Most notably, people who reported that they were using technology for health, had the ability and motivation to engage with digital services, and found that digital services met their individual needs were 3 to 4 times more likely to use or intend to use MyHR compared with their counterparts. Other clear associations included dimensions of health literacy relating to positive relationships with health care providers and social support and using the internet to search for health information.

### Comparison With Prior Work

The limited number of studies on the predictors of PHR uptake and the use of digital health literacy as a general concept in previous studies make it difficult to compare the findings of this study with those of prior studies. Nevertheless, this study found that only one-third of the sample (320/998, 32.1%) was engaged with MyHR, and only a very few participants (63/998, 6.3%) were current users. This is in line with the data revealed at an Australian Senate estimate hearing in December 2019 that only 4% of Australians logged into MyHR more than once [60].

In a qualitative study of 66 Australian women who were regular users of web-based health information, Lupton [61] found that factors such as lack of interest, security, and privacy concerns or not seeing any benefits of using MyHR were potential barriers to using MyHR. This study also identified eHLQ scale 4, *Feel safe and in control,* and scale 5, *Motivated to engage with digital services,* as strongly associated with MyHR awareness, engagement, and use or intention to use. Another notable finding is the eHLQ scale 2, *Understanding of health language and concepts*, which was not associated with awareness but was the strongest predictor of engagement and a moderately strong predictor of use. This finding echoes the October 2021 statistics from MyHR, which showed that the documents most viewed by Australians were pathology reports, with >1.6 million views, a huge jump of 613% compared with the views 12 months earlier [62]. The immunization report, introduced in early 2021, was also one of the top 10 items people looked at in October 2021, with 2.7 million views, an increase of 68% compared with views a month earlier [62]. This could indicate that a better understanding of one’s health is one of the purposes for engaging with MyHR.

### Digital Health Literacy

An important finding of this study is the potential role of digital health literacy in the adoption and use of web-based PHR. Instead of simply reporting that higher or lower digital health literacy is linked to the uptake of PHR, as in the study by Noblin et al [33], this study examined the 7 dimensions of digital health literacy and the relative strengths of the association of digital health literacy with awareness and engagement of PHR. By using a multidimensional instrument, this study provided more nuanced insights into people’s MyHR awareness, engagement, and use or intention to use. The dimensions of using technology for health (eHLQ scale 1, *Using technology to process health information*) and ability (eHLQ scale 3, *Ability to actively engage with digital services*) as well as motivation to use technology (eHLQ scale 5, *Motivated to engage with digital services*) are all strongly associated with using MyHR, with a 4.14- to 4.44-increase in the odds of using or intention to use web-based PHR. This is further confirmed, because using the internet to search for health information was another strong predictor, especially in use or intention to use.

When interpreting the results of this study, it is useful to consider the 7 dimensions of digital health literacy measured and ask the question, “How does a person develop in this dimension?” It is clear that a person’s digital health literacy will develop along with their experiences of using digital health technologies; for better or worse, positive experiences will increase their trust, confidence, and perceptions of value, whereas negative experiences will do the opposite. Therefore, digital health literacy is clearly not just a predictor of the use of technologies but also, if not more so, a consequence, especially among people with few prior experiences of using digital health technologies.

However, the direction of causality was not assumed in this study. Although initiatives to develop digital skills may be an effective way to increase the adoption of MyHR (and is a commonly used strategy to improve digital health literacy [63]), an alternative is to use the assessment of health literacy as a means to understand and shape the experiences of people as they engage with digital health technologies and the processes that build (or undermine) trust, confidence, and perceived benefits. This would lead to a process of designing both the features and rolling out of digital technologies in such a way as to maximize virtuous cycles and minimize confidence eroding cycles, guided by an understanding of the digital health literacy of users. This overall process can be described as “health literacy development.”

### Implications and Recommendations

Although digital skill training may have the potential to increase the likelihood of using web-based PHR and is likely to be practical and easy to implement, it should also be noted that other potential predictors identified in this study may also be important to consider when developing community-based interventions and leveraging the important role that health professionals may have in influencing people’s knowledge and confidence in engaging with digital records. With eHLQ scale 4, *Feel safe and in control,* being a strong predictor, addressing people’s privacy concerns is an action that may be a key building block to engagement. The eHLQ scale 2*, Understanding of health language and concepts*, while was not associated with being aware of MyHR, was moderately associated with engagement and use, suggesting that initiatives to promote the benefits of using the system to better understand and manage people’s own health should be considered. In fact, it is likely that when people see the benefits of using web-based PHR, they may become more motivated to use digital technologies, leading to cyclical growth in both confidence and intent to use MyHR along with digital health literacy.

Besides, the notion of including other nondigital actions is further supported by the finding that predictors of engagement and use included the health literacy dimensions represented by the HLQ scale 1, *Feeling understood and supported by health care providers*, scale 3, *Actively managing my health*, and scale 4, *Social support for health*. This indicates that public health education should not only target individual users of MyHR but also provide supportive health care and social networks to encourage the use of web-based PHR. Hence, an approach that considers the social factors surrounding the use of web-based PHR is a critical aspect of health literacy development.

In a review of the information quality and usability of MyHR in 2018 using a health literacy framework, Walsh et al [64] found that only 16% of such resources could be rated as easy to read, 88% were text based, images to assist learning were limited, and color and large buttons to facilitate engagement and navigation were missing. They concluded that people at risk of lower health literacy did not have equitable access to the system, potentially increasing health disparities between users and nonusers. Although the MyHR website is regularly updated, the MyHR website as of March 2022 features a small button to listen to the website, and translation to other languages is available only for some pages, not the full website. As this study found that digital services that suit individual needs were associated with a 3.5-fold increase in the odds of using or intending to use MyHR, actions such as improving the readability, usability, and accessibility of MyHR to ensure that the system is responsive to all users’ needs may maximize equitable access to web-based PHR. An easy-to-use digital system may boost people’s confidence and motivation to use digital health technologies and, in turn, reinforce their engagement with the system and perhaps develop the digital health literacy of individuals.

Furthermore, this study found that older people and those with less education were less likely to engage in or use MyHR. These findings indicate that there are population groups that may become disadvantaged as countries move to web-based PHR, leading to a potential widening of health disparities. Special initiatives with targeted and tailored interventions are needed to ensure that no group remains left behind. A co-design approach to developing these initiatives with those with lived experience in diverse communities is also recommended, because this approach has been considered the best practice “to reduce inequality and empower vulnerable communities” [65].

### Future Directions

This study was the needs assessment phase of a larger study that used the Ophelia process to identify solutions to maximize the use of digital technologies, including MyHR. The Ophelia process takes a co-design approach to create and implement solutions to understand and improve access, equity, and outcomes by addressing health literacy needs [38]. It has been shown to be effective in various studies [66-69] and is easy to apply in diverse settings from European hospital settings to Egyptian fishing villages [70-73]. In addition to identifying the predictors of PHR use, the data of this study were also used to identify digital health literacy profiles among community members. These profiles reflect the digital health literacy challenges, preferences, and strengths of respondents regarding the adoption and use of MyHR. The next phase of this study involved integrating quantitative data profiles with demographic and interview data to create vignettes (or stories) of typical community members about their use of digital health systems. The vignettes were then presented at idea generation workshops attended by community members and frontline health professionals. This process allows for co-design opportunities using local wisdom to generate clear and actionable recommendations to increase the uptake and use of MyHR and achieve widespread participation in the process of health literacy development.

The finding that only 6.3% of the survey sample were MyHR users indicates that attempts to engage the Australian population with MyHR might not have been successful at that time. Since the opt-out period when around 10% (approximately 2.5 million) of Australians opted out of the system [74], approximately 90,000 people who previously opted out or canceled their records had registered for a record by June 2021 [75]. Further population-based surveys should be undertaken to measure changes in and predictors of awareness, engagement, and use of MyHR, especially as the use of digital health services has become more common during the COVID-19 pandemic. The findings will inform further actions to improve the uptake rate of active MyHR users.

### Limitations

A limitation of this study is that it was conducted in a regional area of Victoria, which may not be representative of the Australian population. However, the aim of this study was to identify digital health literacy needs, and rural residents were found to be among the population groups with greater digital health literacy needs than urban residents [19,21]. It is also noted that there were some demographic groups that were not well represented in the sample when compared with that of residents of the Ballarat Goldfields region; this included a lower representation of younger people and those with lower education. The impact of this discrepancy is that the findings may not be generalizable to the general adult population in the region. However, it is important to note that this study sought to understand people’s experience with digital health services. Given that people in middle to late adulthood have the highest burden of chronic health conditions and tend to have multimorbidity, this population group may have more to gain from the engagement and use of web-based PHR.

Groups experiencing vulnerabilities who are most at risk of digital inequities were also not well represented. Only 1.8% (18/998) of the respondents identified as Aboriginal or Torres Strait Islanders and only 0.8% (8/998) spoke a language other than English at home. Although the proportion of Aboriginal and Torres Strait Islanders was similar to the population of the Ballarat Goldfields region (Multimedia Appendix 2), the proportion of people who spoke a language other than English at home was lower than that in the 2016 Australian Bureau of Statistics Census (39,743/576,802, 6.9%) [76]. Future studies should focus on these 2 groups to better understand their needs and barriers to digital health.

Another limitation is that the sample included only adults who had a landline telephone or mobile phone number registered to a postcode in the Ballarat Goldfields region. Consequently, individuals who did not have access to a phone, who lived in the region but had a phone registered to a different postcode, or who had an unlisted number were not sampled. Such individuals may have had different experiences with health technologies and services than the study sample, and their experiences were not captured in the data. Furthermore, CATI as an administration mode can lead to some cognitive burden and recall bias, which may in turn affect the ways in which people respond [77]. However, the use of a telephone survey is a cost-effective way to undertake a large population survey in a short period.

### Conclusions

This study provides insights into the predictors of the use of a national web-based PHR, MyHR, in Australia, and advances the understanding of the mechanism behind the use of web-based PHRs. These findings suggest that actions to improve the uptake and use of web-based PHRs need to look beyond improving individual digital skills. Of equal importance are initiatives to provide access to digital technologies, develop responsive digital services, provide a better understanding of the benefits of using web-based PHRs, and establish health care and social support networks. Therefore, a holistic approach is essential for enhancing the rate of web-based PHR engagement and use. This study also identified subgroups that are likely to be nonusers of web-based PHRs; targeted solutions need to be put in place to ensure that a web-based PHR can realize its full potential to help reduce health inequities.

## Appendix

Multimedia Appendix 1 eHealth Literacy Questionnaire and Health Literacy Questionnaire scales and definitions.

Multimedia Appendix 2 Comparison of study participants and the population of the Ballarat Goldfields region of the Western Victoria Primary Health Network.

Multimedia Appendix 3 Logistic regression predicting likelihood of My Health Record awareness.

Multimedia Appendix 4 Logistic regression predicting likelihood of My Health Record engagement.

Multimedia Appendix 5 Logistic regression predicting likelihood of My Health Record use or intention to use.

## Abbreviations

CATI: computer-assisted telephone interview
eHLQ: eHealth Literacy Questionnaire
HLQ: Health Literacy Questionnaire
MyHR: My Health Record
Ophelia: Optimizing Health Literacy and Access
OR: odds ratio
PHR: personal health record
WVPHN: Western Victoria Primary Health Network
