# Supplementary material for: Digital Health Literacy as a Predictor of Awareness, Engagement, and Use of a National Web-Based Personal Health Record: Population-Based Survey Study
Source: J Med Internet Res. 2022 Sep 16;24(9):e35772. doi: 10.2196/35772 (PMC9526109; doi:10.2196/35772)
Supplement: Multimedia Appendix 1 [file jmir_v24i9e35772_app1.docx]

## **Multimedia Appendix 1:** eHealth Literacy Questionnaire and Health Literacy Questionnaire scales and definitions.

**eHealth Literacy Questionnaire scales and definitions**

| **1. Using technology to process health information** |
| --- |
| Be able to use technologies to read, write and remember, apply basic numerical concepts, and understand context-specific language (e.g. health, IT or English) as well as critically appraise information. Know when, how and what information to use. |
| **2. Understanding of health concepts and language** |
| Know about basic physiological functions and own current health status. Aware of risk factors and how to avoid them or reduce their influence on own health. |
| **3. Ability to actively engage with digital services** |
| Being comfortable using digital services for handling information. |
| **4. Feel safe and in control** |
| Feel that they have the ownership of personal data stored in the systems and that the data are safe and can be accessed only by people to whom they are relevant (own doctor/nurse etc.). |
| **5. Motivated to engage with digital services** |
| Feel that engaging in the use of digital services will be useful for them in managing their health. |
| **6. Access to digital services that work** |
| Have access to digital services that the users trust to be working when they need it and as they expect it to work. |
| **7. Digital services that suit individual needs** |
| Have access to digital services that suit the specific needs and preferences of the users. This includes responsive features of both IT and the healthcare system (including carers) as well as adaptation of devices and interfaces to be used by people with physical and mental disabilities. |

**Health Literacy Questionnaire scales and definitions**

| **1. Feeling understood and supported by healthcare providers** |
| --- |
| **High:** Has an established relationship with at least one healthcare provider who knows them well and who they trust to provide useful advice and information and to assist them to understand information and make decisions about their health. |
| **Low:** People are unable to engage with doctors and other healthcare providers. They don’t have a regular healthcare provider and/or have difficulty trusting healthcare providers as a source of information and/or advice. |
| **2. Having sufficient information to manage my health** |
| **High:** Feels confident that they have all the information that they need to live with and manage their condition and to make decisions. |
| **Low:** Feels that there are many gaps in their knowledge and that they don't have the information they need to live with and manage their health concerns. |
| **3. Actively managing my health** |
| **High:** Recognise the importance of and are able to take responsibility for their own health. They proactively engage in their own care and make their own decisions about their health. |
| **Low:** People don’t see their health as their responsibility, they are not engaged in their healthcare and regard healthcare as something that is done to them. |
| **4. Social support for health** |
| **High:** A person’s social system provides them with all the support they want or need. |
| **Low:** Completely alone and unsupported. |
| **5. Appraisal of health information** |
| **High:** Able to identify good information and reliable sources of information. They can resolve conflicting information by themselves or with help from others. |
| **Low:** No matter how hard they try, they cannot understand most health information and get confused when there is conflicting information. |
| **6. Ability to actively engage with healthcare providers** |
| **High:** Is proactive about their health and feels in control in relationships with healthcare providers. Is able to seek advice from additional health care providers when necessary. They keep going until they get what they want. Empowered. |
| **Low:** Is passive in their approach to health care, inactive, i.e., they do not proactively seek or clarify information and advice and/or service options. They accept information without question. Unable to ask questions to get information or to clarify what they don’t understand. They accept what is offered without seeking to ensure that it meets their needs. Feel unable to share concerns. |
| **7. Navigating the healthcare system** |
| **High:** Able to find out about services and supports so they get all their needs met. Able to advocate on their own behalf at the system and service level. |
| **Low:** Unable to advocate on their own behalf and unable to find someone who can help them use the healthcare system to address their health needs. Do not look beyond obvious resources and have a limited understanding of what is available and what they are entitled to. |
| **8. Ability to find good health information** |
| **High:** Is an 'information explorer'. Actively uses a diverse range of sources to find information and is up to date. |
| **Low:** Cannot access health information when required. Is dependent on others to offer information. |
| **9. Understand health information well enough to know what to do** |
| **High:** Is able to understand all written information (including numerical information) in relation to their health and able to write appropriately on forms where required |
| **Low:** Has problems understanding any written health information or instructions about treatments or medications. Unable to read or write well enough to complete medical forms |
