# Supplementary material for: Digital Health Literacy as a Predictor of Awareness, Engagement, and Use of a National Web-Based Personal Health Record: Population-Based Survey Study
Source: J Med Internet Res. 2022 Sep 16;24(9):e35772. doi: 10.2196/35772 (PMC9526109; doi:10.2196/35772)
Supplement: Multimedia Appendix 2 [file jmir_v24i9e35772_app2.docx]

## **Multimedia Appendix 2:** Comparison of study participants and the population of Ballarat Goldfields region of the Western Victoria Primary Health Network.

|  | **Computer Assisted Telephone Interview** (**CATI) sample (%)** | **Western Victoria Primary Health Network^a^ (%)** |
| --- | --- | --- |
| **Sex** |  |  |
| Female | 53.7 | 51.2 |
| Male | 46.3 | 48.8 |
| **Age** |  |  |
| <55 years | 20.2 | 67.3 |
| 55 to 70 | 38.5 | 19.9 |
| >70 years | 41.2 | 12.8 |
| **Aboriginal or Torres Strait Islander** |  | |
| Yes | 1.8 | 1.4 |
| No/Not stated | 98.2 | 98.6 |
| **Highest educational attainment** |  | |
| Secondary school or below | 44.5 | 42.3 |
| Trade, apprenticeship, certificate or diploma | 24.6 | 26.8 |
| University | 30.8 | 17 |
| Not stated | - | 11.8 |

^a^N=157,572, Source: Australian Government Department of Health (04 May 2018). "Primary Health Networks: Demographic Data 2018." Retrieved 8th January, 2019, from <http://www.health.gov.au/internet/main/publishing.nsf/Content/PHN-Demographic_Data>.
