# Supplementary material for: Digital Health Literacy as a Predictor of Awareness, Engagement, and Use of a National Web-Based Personal Health Record: Population-Based Survey Study
Source: J Med Internet Res. 2022 Sep 16;24(9):e35772. doi: 10.2196/35772 (PMC9526109; doi:10.2196/35772)
Supplement: Multimedia Appendix 3 [file jmir_v24i9e35772_app3.docx]

## **Multimedia Appendix 3:** Logistic regression predicting likelihood of My Health Record awareness.^a,b^

|  |  | **Unaware** | **Aware** |  |  |
| --- | --- | --- | --- | --- | --- |
|  | N | mean or proportion  (95% CI) N=359 | mean or proportion  (95% CI) N=639 | Odds Ratio  (95% CI) | *P* value |
| **Sex** |  |  |  |  |  |
| Male | 462 | 40.5 (31.6, 45) | 59.5 (55, 63.9) | ref^c^ |  |
| Female | 536 | 32.1 (28.3, 36.2) | 67.9 (63.8, 71.7) | 1.44 (1.11, 1.87) | .01 |
| **Age** |  |  |  |  |  |
| Years of age (continuous) | 998 | 64.3 (62.8, 65.8) | 65.2 (64.2, 66.3) | 1.01 (1.00, 1.02) | .28 |
| **Highest educational attainment** | | |  |  |  |
| Did not complete secondary school | 309 | 36.8 (31.7, 42.4) | 63.1 (57.6, 68.3) | ref |  |
| Completed secondary school | 136 | 36 (28.4, 44.4) | 64 (55.6, 71.6) | 1.09 (0.71, 1.66) | .71 |
| Trade, apprenticeship, certificate or diploma | 246 | 35.8 (30, 42) | 64.2 (58, 70) | 1.09 (0.76, 1.54) | .65 |
| University | 307 | 35.2 (30, 40.7) | 64.8 (59.3, 70) | 1.12 (0.80, 1.56) | .51 |
| **Number of long-standing conditions** | | | | | |
| No conditions | 585 | 36.6 (32.8, 40.6) | 63.4 (59.4, 67.2) | ref |  |
| 1 condition | 272 | 32.7 (27.4, 38.5) | 67.2 (61.4, 72.6) | 1.16 (0.85, 1.57) | .77 |
| 2 or more conditions | 141 | 39.7 (32, 48) | 60.2 (52, 68) | 0.85 (0.58, 1.24) | .59 |
| **Self-rated health** | |  |  |  |  |
| Excellent | 170 | 35.9 (29, 43.4) | 64.1 (56.6, 71) | ref |  |
| Very good | 270 | 37 (31.5, 43) | 63 (57, 68.5) | 0.94 (0.63, 1.41) | .77 |
| Good | 293 | 34.8 (29.6, 40.5) | 65.2 (59.5, 70.4) | 1.04 (0.70, 1.54) | .87 |
| Fair | 165 | 32.7 (26, 40.4) | 67.2 (59.7, 74) | 1.12 (0.71, 1.77) | .62 |
| Poor or Very Poor | 100 | 42 (32.7, 51.9) | 58 (48.1, 67.2) | 0.76 (0.46, 1.27) | .30 |
| **Use of the internet to access health-related information** | | | |  |  |
| No, or NA | 420 | 40 (35.6, 45) | 59.8 (55, 64.4) | ref |  |
| Yes | 578 | 32.9 (29.2, 36.8) | 67.1 (63.2, 70.8) | 1.52 (1.15, 2.01) | .003 |
| **Number of contacts with a health professional over the past 12 months** | | | | |  |
| 13 or more | 269 | 34.6 (29.1, 40.5) | 6.45 (59.5, 70.9) | ref |  |
| 7 to 12 | 196 | 36.7 (30.3, 43.7) | 63.3 (56.3, 69.7) | 0.91 (0.62, 1.34) | .65 |
| 2 to 6 | 446 | 36.1 (31.8, 40.7) | 63.9 (59.3, 68.2) | 0.94 (0.69, 1.29) | .71 |
| 0 to 1 | 87 | 37.9 (28.4, 48.5) | 62.1 (51.5, 71.6) | 0.90 (0.54, 1.49) | .68 |
| **eHealth Literacy Questionnaire scales (range 1.00 to 4.00)** | | | |  |  |
| **1. Using technology to process health information** | | | |  |  |
|  | | 2.21 (2.15, 2.28) | 2.41 (2.37, 2.46) | 1.77 (1.42, 2.22) | <.001 |
| **2. Understanding of health concepts and language** | | | |  |  |
|  | | 2.91 (2.87, 2.95) | 2.94 (2.92, 3.00) | 1.28 (0.92, 1.77) | .14 |
| **3. Ability to actively engage with digital services** | | | | | |
|  | | 2.35 (2.27, 2.42) | 2.52 (2.5-, 2.56) | 1.53 (1.25, 1.89) | <.001 |
| **4. Feel safe and in control** | | | |  |  |
|  | | 2.51 (2.46, 2.58) | 2.64 (2.60, 2.68) | 1.47 (1.17, 1.85) | .001 |
| **5. Motivated to engage with digital services** | | | | | |
|  | | 2.30 (2.21, 2.35) | 2.47 (2.43, 2.51) | 1.74 (1.40, 2.19) | <.001 |
| **6. Access to digital services that work** | | | | | |
|  | | 2.41 (2.36, 2.47) | 2.57 (2.53, 2.61) | 1.99 (1.51, 2.63) | <.001 |
| **7. Digital services that suit individual needs** | | | | | |
|  | | 2.28 (2.21, 2.35) | 2.44 (2.40, 2.49) | 1.63 (1.30, 2.04) | <.001 |
| **Health Literacy Questionnaire scales (range 1.00 to 4.00)** | | | | | |
| **1. Feeling understood and supported by healthcare providers** | | | | | |
|  | | 3.14 (3.08, 3.19) | 3.17 (3.14, 3.22) | 1.15 (0.90, 1.48) | .26 |
| **3. Actively managing my health** | | | | | |
|  | | 3.03 (2.99, 3.08) | 3.01 (3.00, 3.04) | 0.89 (0.66, 1.20) | .46 |
| **4. Social support for health** | | | | | |
|  | | 3.04 (2.99, 3.10) | 3.05 (3.01, 3.09) | 1.02 (0.78, 1.33) | .89 |
| **Health Literacy Questionnaire scale (range 1.00 to 5.00)** | | | | | |
| **7. Navigating the healthcare system** | | | | | |
|  | | 3.91 (3.84, 4.00) | 3.96 (3.91, 4.02) | 1.10 (0.92, 1.31) | .29 |

^a^Analyses were adjusted for age.

^b^Participants were asked “Do you have a My Health Record”; those who responded “Not sure” were characterized as “Unaware” (N=639), those who responded either “Yes” or “No” were characterized as “Aware” (N=359).

^c^ref = reference subgroup.

##
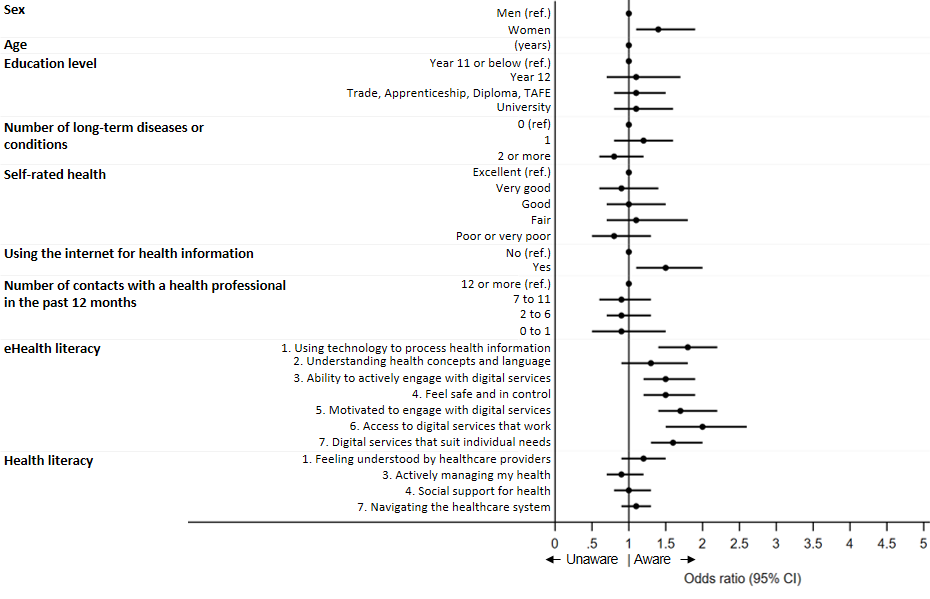
Forest plot summary of logistic regression predicting likelihood of My Health Record (MyHR) awareness.^a^

^a^Participants were asked “Do you have a My Health Record”; those who responded “Not sure” were characterized as “Unaware” (N=639), those who responded either “Yes” or “No” were characterized as “Aware” (N=359); ref = reference subgroup; analyses were adjusted for age.
