# Supplementary material for: Digital Health Literacy as a Predictor of Awareness, Engagement, and Use of a National Web-Based Personal Health Record: Population-Based Survey Study
Source: J Med Internet Res. 2022 Sep 16;24(9):e35772. doi: 10.2196/35772 (PMC9526109; doi:10.2196/35772)
Supplement: Multimedia Appendix 4 [file jmir_v24i9e35772_app4.docx]

## **Multimedia Appendix 4:** Logistic regression predicting likelihood of My Health Record engagement.^a,b^

|  |  | **Not engaged** | **Engaged** |  |  |
| --- | --- | --- | --- | --- | --- |
|  | N | mean or proportion  (95% CI) n = 319 | mean or proportion  (95% CI) n = 320 | Odds Ratio  (95% CI) | *P* value |
| **Sex** |  |  |  |  |  |
| Male | 275 | 47.6 (421.8, 53.6) | 52.4 (46.4, 58.2) | ref^c^ |  |
| Female | 364 | 51.6 (46.5, 56.8) | 48.4 (43.2, 53.5) | 0.85 (0.62, 1.17) | .32 |
| **Age** |  |  |  |  |  |
| Years of age (continuous) | 639 | 67.2 (65.8, 68.6) | 63.3 (61.8, 64.8) | 0.98 (0.97, 0.99) | <.001 |
| **Highest educational attainment** | | |  |  |  |
| Did not complete secondary school | 195 | 56.7 (49.9, 63.7) | 43.1 (36.3, 50.1) | ref |  |
| Completed secondary school | 87 | 50.6 (40.2, 60.9) | 49.4 (39.1, 59.8) | 1.09 (0.65, 1.83) | .75 |
| Trade, apprenticeship, certificate or diploma | 158 | 49.4 (41.6, 57.1) | 50.6 (42.9, 58.4) | 1.20 (0.78, 1.85) | .40 |
| University | 199 | 43.2 (36.5, 50.2) | 56.8 (49.8, 63.5) | 1.50 (0.99, 2.26) | .05 |
| **Number of long-standing conditions** | | | |  |  |
| No conditions | 371 | 53.6 (48.5, 58.7) | 46.47 (41.3, 51.5) | ref |  |
| 1 condition | 183 | 46.5 (39.3, 53.7) | 53.6 (46.3, 60.7) | 1.48 (1.03, 2.13) | .30 |
| 2 or more conditions | 85 | 41.2 (31.2, 51.9) | 58.8 (48.1, 68.8) | 1.88 (1.16, 3.07) | .006 |
| **Self-rated health** | |  |  |  |  |
| Excellent | 109 | 52.3 (42.9, 61.5) | 47.7 (38.5, 57.0) | ref |  |
| Very good | 170 | 47.6 (40.2, 55.2) | 52.4 (44.8, 59.8) | 1.24 (0.76, 2.02) | .38 |
| Good | 191 | 49.7 (42.7, 56.8) | 50.3 (43.2, 57.3) | 1.20 (0.74, 1.93) | .46 |
| Fair | 111 | 47.7 (38.6, 57.0) | 52.3 (43.0, 61.4) | 1.33 (0.77, 2.27) | .31 |
| Poor or Very Poor | 58 | 56.9 (44.0, 69.0) | 43.1 (31.0, 56.1) | 0.88 (0.46, 1.69) | .71 |
| **Use of the internet to access health-related information** | | | |  |  |
| No, or NA | 251 | 61.0 (54.8, 66.8) | 39.0 (33.2, 45.2) | ref |  |
| Yes | 388 | 42.8 (38.0, 47.8) | 57.2 (52.2, 62.1) | 1.81 (1.28, 2.55) | .001 |
| **Number of contacts with a health professional over the past 12 months** | | | | |  |
| 13 or more | 176 | 46.0 (38.8, 53.4) | 54.0 (46.6, 61.2) | ref |  |
| 7 to 12 | 124 | 46.0 (37.4, 54.8) | 54.0 (45.2, 62.6) | 1.00 (0.63, 1.60) | 1.00 |
| 0 to 6 | 339 | 53.4 (48.1, 58.7) | 46.6 (41.3, 52.0) | 0.70 (0.48, 1.02) | .06 |
| **eHealth Literacy Questionnaire scales (range 1.00 to 4.00)** | | | |  |  |
| **1. Using technology to process health information** | | | |  |  |
|  | | 2.33 (2.28, 2.39) | 2.50 (2.44, 2.56) | 1.81 (1.35, 2.42) | <.001 |
| **2. Understanding of health concepts and language** | | | |  |  |
|  | | 2.88 (2.84, 2.92) | 3.02 (2.98, 3.06) | 2.62 (1.70, 4.02) | <.001 |
| **3. Ability to actively engage with digital services** | | | |  |  |
|  | | 2.39 (2.33, 2.45) | 2.64 (2.58, 2.71) | 2.12 (1.60, 2.81) | <.001 |
| **4. Feel safe and in control** | | | |  |  |
|  | | 2.57 (2.51, 2.63) | 2.71 (2.65, 2.77) | 1.61 (1.20, 2.14) | .001 |
| **5. Motivated to engage with digital services** | | | |  |  |
|  | | 2.37 (2.32, 2.43) | 2.57 (2.51, 2.63) | 2.00 (1.48, 2.71) | <.001 |
| **6. Access to digital services that work** | | | |  |  |
|  | | 2.51 (2.46, 2.55) | 2.63 (2.59, 2.68) | 1.90 (1.33, 2.70) | <.001 |
| **7. Digital services that suit individual needs** | | | |  |  |
|  | | 2.35 (2.29, 2.41) | 2.53 (2.48, 2.59) | 1.89 (1.40, 2.55) | <.001 |
| **Health Literacy Questionnaire scales (range 1.00 to 4.00)** | | | |  |  |
| **1. Feeling understood and supported by healthcare providers** | | | |  |  |
|  | | 3.11 (3.06, 3.17) | 3.24 (3.19, 3.29) | 1.63 (1.19, 2.22) | .002 |
| **3. Actively managing my health** | | | |  |  |
|  | | 3.00 (2.94, 3.04) | 3.03 (2.98, 3.08) | 1.25 (0.87, 1.80) | .24 |
| **4. Social support for health** | | | |  |  |
|  | | 2.98 (2.93, 3.04) | 3.11 (3.06, 3.16) | 1.74 (1.25, 2.42) | .001 |
| **Health Literacy Questionnaire scale (range 1.00 to 5.00)** | | | |  |  |
| **7. Navigating the healthcare system** | | | |  |  |
|  | | 3.93 (3.85, 4.01) | 4.00 (3.92, 4.08) | 1.15 (0.93, 1.42) | .20 |

^a^Analyses were adjusted for age.

^b^Participants were asked “Do you have a My Health Record”: those who responded “No” were characterized as “Not engaged” (N=319); those who responded “Yes” were characterized as “Engaged” (N=320).

^c^ref = reference subgroup.

## Forest plot summary of logistic regression predicting likelihood of My Health Record (MyHR) engagement.^a^


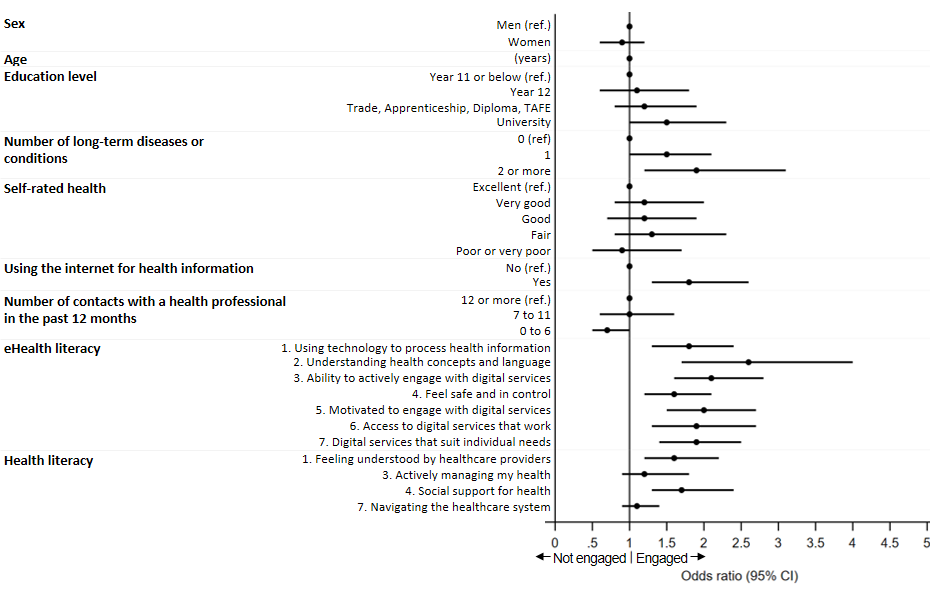


^a^Participants were asked “Do you have a My Health Record”: those who responded “No” were characterized as “Not engaged” (N=319); those who responded “Yes” were characterized as “Engaged” (N=320); ref = reference subgroup; analyses were adjusted for age.
