# Supplementary material for: Digital Health Literacy as a Predictor of Awareness, Engagement, and Use of a National Web-Based Personal Health Record: Population-Based Survey Study
Source: J Med Internet Res. 2022 Sep 16;24(9):e35772. doi: 10.2196/35772 (PMC9526109; doi:10.2196/35772)
Supplement: Multimedia Appendix 5 [file jmir_v24i9e35772_app5.docx]

## **Multimedia Appendix 5:** Logistic regression predicting likelihood of My Health Record use or intention to use.^a,b^

|  |  | **Non-user** | **User** |  |  |
| --- | --- | --- | --- | --- | --- |
|  | N | mean or proportion  (95% CI) n = 86 | mean or proportion  (95% CI) n = 166 | Odds Ratio  (95% CI) | *P* value |
| **Sex** |  |  |  |  |  |
| Male | 117 | 41.0 (32.5, 50.2) | 59.0 (50.0, 67.5) | ref^c^ |  |
| Female | 135 | 28.1 (21.2, 36.3) | 71.9 (63.7, 78.8) | 1.78 (1.05, 3.00) | <.001 |
| **Age** |  |  |  |  |  |
| Years of age (continuous) | 252 | 65.7 (63.2, 68.3) | 62.2 (60.1, 64.3) | 0.98 (0.96, 1.00) | .10 |
| **Highest educational attainment** | | |  |  |  |
| Did not complete secondary school | 71 | 45.1 (33.9, 56.7) | 54.9 (43.3, 66.1) | ref |  |
| Completed secondary school or trade, apprenticeship, certificate or diploma | 93 | 36.6 (27.4, 46.8) | 63.4 (53.2, 72.6) | 1.32 (0.69, 2.50) | .40 |
| University | 88 | 22.7 (15.1, 32.7) | 77.3 (67.4, 84.9) | 2.48 (1.23, 5.02) | .01 |
| **Number of long-standing conditions** | | | |  |  |
| No conditions | 138 | 31.2 (24.0, 39.4) | 68.9 (60.6, 76.0) | ref |  |
| 1 or more condition | 114 | 37.7 (29.3, 47.0) | 62.3 (53.0, 70.7) | 0.85 (0.49, 1.46) | .10 |
| **Self-rated health** | |  |  |  |  |
| Excellent or very good | 109 | 31.2 (23.2, 40.5) | 68.8 (59.5, 76.8) | ref |  |
| Good, fair, poor, or very poor | 143 | 36.4 (28.9, 44.6) | 63.6 (55.4, 71.1) | 0.86 (0.50, 1.48) | .60 |
| **Use of the internet to access health-related information** | | | |  |  |
| No, or NA | 78 | 52.6 (41.5, 63.4) | 47.4 (36.6, 58.5) | ref |  |
| Yes | 174 | 25.9 (19.9, 32.9) | 74.1 (67.1, 80.1) | 2.96 (1.64, 5.37) | <.001 |
| **Number of contacts with a health professional over the past 12 months** | | | | |  |
| 7 or more | 125 | 31.2 (23.7, 40.0) | 68.8 (60.1, 76.3) | ref |  |
| 0 to 6 | 127 | 37.0 (29.0, 45.8) | 63.0 (54.3, 71.0) | 0.71 (0.41, 1.20) | .20 |
| **eHealth Literacy Questionnaire scales (range 1.00 to 4.00)** | | | | |  |
| **1. Using technology to process health information** | | | |  |  |
|  | | 2.31 (2.21, 2.41) | 2.68 (2.60, 2.76) | 4.14 (2.34, 7.31) | <.001 |
| **2. Understanding of health concepts and language** | | | |  |  |
|  | | 2.96 (2.89, 3.03) | 3.07 (3.01, 3.13) | 2.25 (1.08, 4.69) | .03 |
| **3. Ability to actively engage with digital services** | | | |  |  |
|  | | 2.38 (2.26, 2.50) | 2.81 (2.73, 2.89) | 4.44 (2.55, 7.75) | <.001 |
| **4. Feel safe and in control** | | | |  |  |
|  | | 2.57 (2.44, 2.69) | 2.82 (2.74, 2.89) | 2.36 (1.43, 3.88) | .001 |
| **5. Motivated to engage with digital services** | | | |  |  |
|  | | 2.38 (2.28, 2.49) | 2.74 (2.69, 2.82) | 4.24 (2.36, 7.61) | <.001 |
| **6. Access to digital services that work** | | | |  |  |
|  | | 2.55 (2.46, 2.65) | 2.72 (2.66, 2.79) | 2.49 (1.32, 4.69) | .01 |
| **7. Digital services that suit individual needs** | | | |  |  |
|  | | 2.36 (2.25, 2.48) | 2.67 (2.60, 2.75) | 3.48 (1.97, 6.15) | <.001 |
| **Health Literacy Questionnaire scales (range 1.00 to 4.00)** | | | |  |  |
| **1. Feeling understood and supported by healthcare providers** | | | |  |  |
|  | | 3.17 (3.07, 3.26) | 3.32 (3.24, 3.40) | 1.89 (1.10, 3.27) | .02 |
| **3. Actively managing my health** | | | |  |  |
|  | | 2.97 (2.89, 3.05) | 3.11 (3.04, 3.18) | 2.28 (1.18, 4.38) | .01 |
| **4. Social support for health** | | | |  |  |
|  | | 3.03 (2.93, 3.13) | 3.17 (3.11, 3.24) | 2.10 (1.15, 3.84) | .02 |
| **Health Literacy Questionnaire scale (range 1.00 to 5.00)** | | | | |  |
| **7. Navigating the healthcare system** | | |  |  |  |
|  | | 3.92 (3.73, 4.10) | 4.03 (3.93, 4.13) | 1.24 (0.87, 1.75) | .23 |

^a^Analyses were adjusted for age.

^b^Participants who were asked “Do you have a My Health Record”; those who responded “Yes” were then asked whether they use or intend to use their My Health Record; those who responded “No” were characterised as “Non-users” (N=86), those who responded that they currently use My Health Record or intended to use the My Health Record were characterised as “Users” (N=166).

^c^ref = reference subgroup.

## Forest plot summary of logistic regression predicting likelihood of My Health Record (MyHR) use or intention to use.^a^


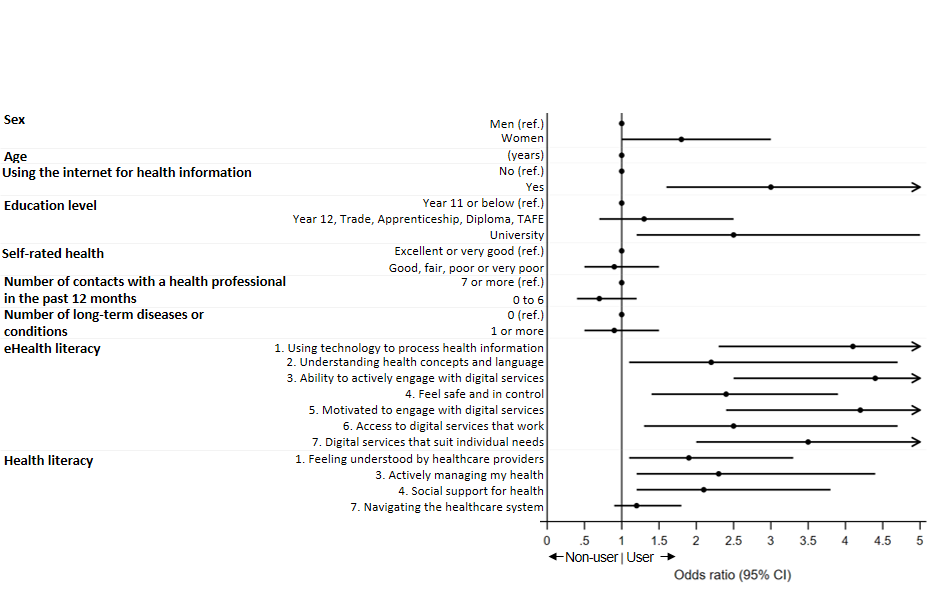


^a^Participants who were asked “Do you have a My Health Record”; those who responded “Yes” were then asked whether they use or intend to use their My Health Record; those who responded “No” were characterized as “Non-users” (N=86), those who responded that they either currently use My Health Record or intended to were characterized as “User” (N=166); ref = reference subgroup; analyses were adjusted for age.
